# Evaluation of human-baited double net trap and human-odour-baited CDC light trap for outdoor host-seeking malaria vector surveillance in Kenya and Ethiopia

**DOI:** 10.1186/s12936-020-03244-2

**Published:** 2020-05-07

**Authors:** Teshome Degefa, Delenasaw Yewhalaw, Guofa Zhou, Harrysone Atieli, Andrew K. Githeko, Guiyun Yan

**Affiliations:** 1 School of Medical Laboratory Sciences, Faculty of Health Sciences, Jimma University, Jimma, Ethiopia; 2 Centre for Global Health Research, Kenya Medical Research Institute, Kisumu, Kenya; 3 Tropical and Infectious Diseases Research Center (TIDRC), Jimma University, Jimma, Ethiopia; 4 Program in Public Health, College of Health Sciences, University of California at Irvine, Irvine, CA 92697 USA; 5 School of Public Health and Community Development, Maseno University, Kisumu, Kenya

**Keywords:** Malaria vectors, Outdoor host-seeking, Surveillance, Human-odour-baited CDC light trap, Human-baited double net trap, Kenya, Ethiopia

## Abstract

**Background:**

Surveillance of outdoor host-seeking malaria vectors is crucial to monitor changes in vector biting behaviour and evaluate the impact of vector control interventions. Human landing catch (HLC) has been considered the most reliable and gold standard surveillance method to estimate human-biting rates. However, it is labour-intensive, and its use is facing an increasing ethical concern due to potential risk of exposure to infectious mosquito bites. Thus, alternative methods are required. This study was conducted to evaluate the performance of human-odour-baited CDC light trap (HBLT) and human-baited double net trap (HDNT) for outdoor host-seeking malaria vector surveillance in Kenya and Ethiopia.

**Methods:**

The sampling efficiency of HBLT and HDNT was compared with CDC light trap and HLC using Latin Square Design in Ahero and Iguhu sites, western Kenya and Bulbul site, southwestern Ethiopia between November 2015 and December 2018. The differences in *Anopheles* mosquito density among the trapping methods were compared using generalized linear model.

**Results:**

Overall, 16,963 female *Anopheles* mosquitoes comprising *Anopheles gambiae* sensu lato (s.l.), *Anopheles funestus* s.l., *Anopheles pharoensis*, *Anopheles coustani* and *Anopheles squamosus* were collected. PCR results (n = 552) showed that *Anopheles arabiensis* was the only member of *An. gambiae* s.l. in Ahero and Bulbul, while 15.7% *An. arabiensis* and 84.3% *An. gambiae* sensu stricto (s.s.) constituted *An. gambiae* s.l. in Iguhu. In Ahero, HBLT captured 2.23 times as many *An. arabiensis* and 2.11 times as many *An. funestus* as CDC light trap. In the same site, HDNT yielded 3.43 times more *An. arabiensis* and 3.24 times more *An. funestus* than HBLT. In Iguhu, the density of *Anopheles* mosquitoes did not vary between the traps (p > 0.05). In Bulbul, HBLT caught 2.19 times as many *An. arabiensis* as CDC light trap, while HDNT caught 6.53 times as many *An. arabiensis* as CDC light trap. The mean density of *An. arabiensis* did not vary between HDNT and HLC (p = 0.098), whereas the HLC yielded significantly higher density of *An. arabiensis* compared to HBLT and CDC light trap. There was a significant density-independent positive correlation between HDNT and HLC (r = 0.69).

**Conclusion:**

This study revealed that both HBLT and HDNT caught higher density of malaria vectors than conventional CDC light trap. Moreover, HDNT yielded a similar vector density as HLC, suggesting that it could be an alternative tool to HLC for outdoor host-seeking malaria vector surveillance.

## Background

Estimating the entomological inoculation rate (EIR), the number of infectious mosquito bites per person per unit time, is a key metric used to quantify malaria transmission intensity and evaluate the impact of vector control interventions [1, 2]. Estimating EIR requires sampling host-seeking *Anopheles* mosquitoes to determine human-biting rate (HBR) and sporozoite infection rate, the two components of the EIR [1, 3]. However, developing standardized methods for estimating the HBR that do not expose collectors to infectious mosquito bites has been a major challenge [4, 5], especially in settings where a substantial proportion of biting occurs outdoors [6–9].

The gold standard method to determine the HBR has been the human landing catch (HLC), which can be employed either indoors or outdoors to capture mosquitoes as they land to feed on a human host [4, 10–12]. However, HLC is a labour-intensive procedure requiring highly trained collectors and extensive supervision to obtain reliable results. Furthermore, there may be considerable differences between biting rates experienced by different collectors as a result of variability in individual attractiveness and skill in catching mosquitoes [13–15], thus it might be difficult to standardize the estimates based on biting catches. Last but not the least, conducting HLC raises ethical concerns associated with an increased risk of participants’ exposure to infectious mosquito bites if an appropriate anti-malarial chemoprophylaxis is not taken [4, 10, 16]. The increasing risk of arboviral infections further compounds its limitations [17]. Hence, it may not be practical to deploy HLC for routine malaria vector surveillance.

As an alternative to HLC, Centers for Disease Control and Prevention (CDC) miniature light trap has been widely employed for host-seeking mosquito collection [18–20]. The CDC light trap has been shown to have a good performance when used indoors due to its proximity to a sleeping person underneath a bed net [18, 21–23] and has been used as a proxy to estimate indoor-HBRs in different settings [24–26]. However, it may not be effective for the surveillance of outdoor biting malaria vectors in the absence of additional attractants that augment its trapping efficiency [20, 27, 28].

Consequently, efforts have been made to develop and evaluate alternative odour-baited trapping methods in a variety of settings for determining outdoor-HBRs. These include double bed-net traps [29–31], tent traps [32–35] and Mbita traps [36]. The double net traps have been shown to have good efficiency when compared to HLC in some settings [29, 30]. However, they do have their own drawbacks. In some studies, for instance, two persons are used to conduct a double net trap i.e. one individual acting as a bait and the other as collector, and such approach is almost as labour intensive as conducting HLC [30]. In another circumstance when one person is used both as bait and collector [29], there might be a possibility of exposure to infectious mosquito bites during the collection process. A similar concern related with operator’s exposure to mosquito bites has also been reported for the tent traps, despite their promising potential for monitoring host-seeking malaria vectors [32]. Although the Mbita trap is considered an exposure-free tool, it is less effective compared to both HLC and CDC light trap [36–38]. Hence, there is a need to look for an appropriate tool that is as effective as HLC outdoors, exposure-free and widely deployable.

The aim of this study was to evaluate the performance of two novel, exposure-free traps i.e. human-odour-baited CDC light trap (HBLT) and human-baited double net trap (HDNT) for outdoor host-seeking malaria vector surveillance. The HBLT consists of a CDC light trap baited with human-odour pumped from ordinary sleeping room, whereas the HDNT is a variant of previously designed double net trap [29] with an integrated CDC light trap. The trapping efficiency of the HBLT and HDNT was compared with conventional (unbaited) CDC light trap and HLC in western Kenya and southwestern Ethiopia. These study locations were chosen to evaluate the traps in diverse eco-epidemiological settings.

## Methods

### Study sites

**Fig. 1:**
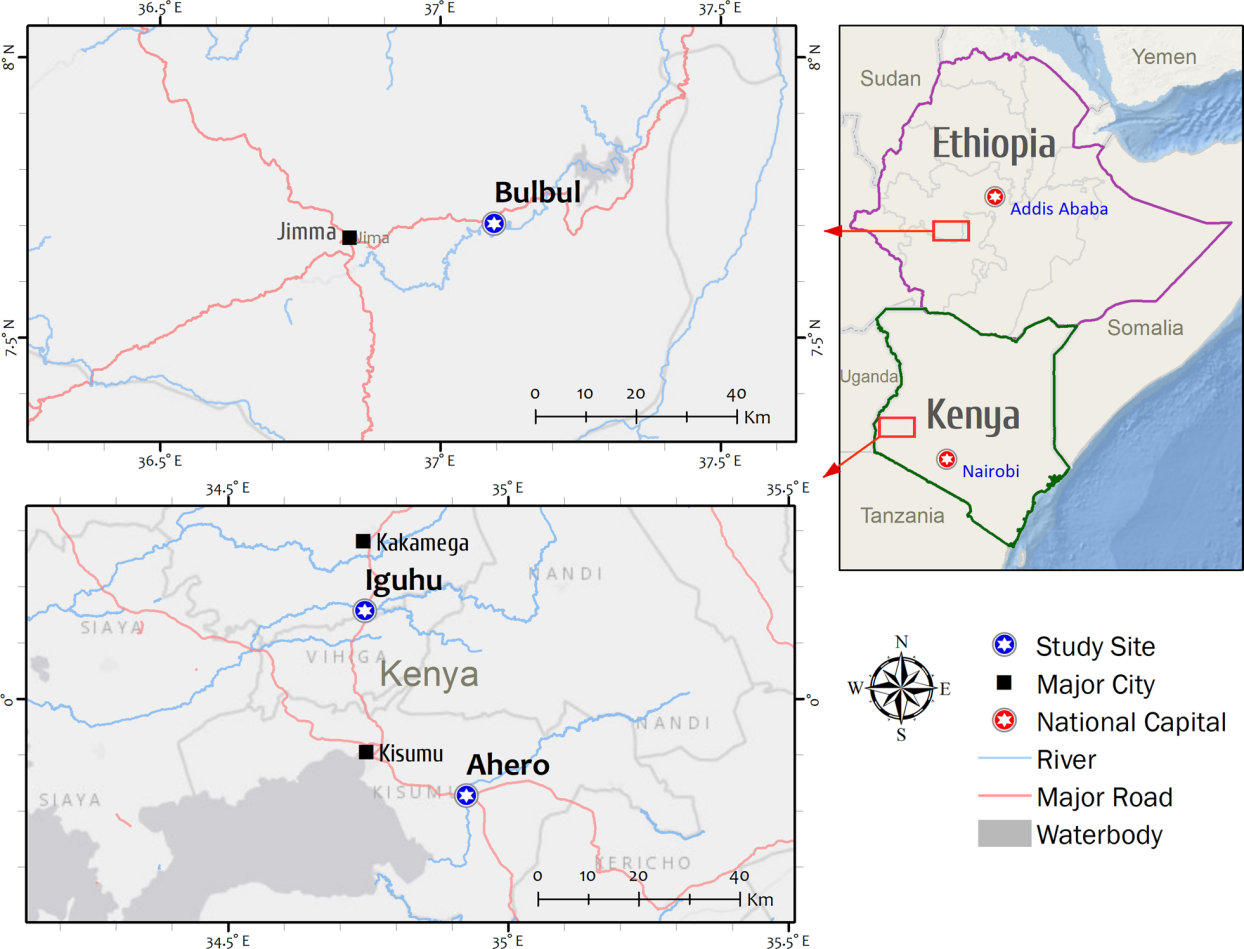
Map of the study sites

The study was conducted in two different eco-epidemiological settings of East Africa, western Kenya and southwestern Ethiopia (Fig. 1).

#### Western Kenya

The study was done in Ahero (0.13123° S, 34.93960° E, altitude 1162 m above sea level, asl) and Iguhu (0.15657° N; 34.74386° E, altitude 1430–1580 m asl) sites. Ahero is a lowland plain area located in Kisumu County while Iguhu is highland site characterized by undulating hills and valley bottoms located in Kakamega County [39, 40]. In both sites, most houses are mud-walled with roofs made of corrugated iron sheets. The inhabitants mainly depend on subsistence farming, with rice and maize being the main cultivated crops in the area. The sites have bimodal pattern of rainfall, with a long rainy season from April to June, which triggers the peak malaria transmission and short rains from October to November with minor transmission [41]. *Plasmodium falciparum* is the predominant malaria parasite species in the area and transmitted by *Anopheles gambiae* sensu stricto (s.s.)*, Anopheles arabiensis* and *Anopheles funestus* [39, 42–44].

#### Southwestern Ethiopia

The study was carried out in Bulbul *kebele* (7.70285° N; 37.09592° E, altitude 1705 m asl), which is located in Kersa district, Oromia Region at 320 kms southwest of Addis Ababa. The majority of the houses are mud-walled with roofs made of corrugated iron sheets. The inhabitants mostly rely on subsistence farming. Maize and *teff* are the main cultivated crops. Malaria transmission is seasonal in Bulbul area. The transmission peaks from September to October, following the major rains from June to September. Minor transmission occurs in April and May, following the short rains of February to March. *Plasmodium falciparum* and *Plasmodium vivax* are the two predominant malaria parasite species in the area and are transmitted by *An. arabiensis* [45].

### Description of trapping methods

#### Human-odour baited CDC light traps (HBLT)

**Fig. 2:**
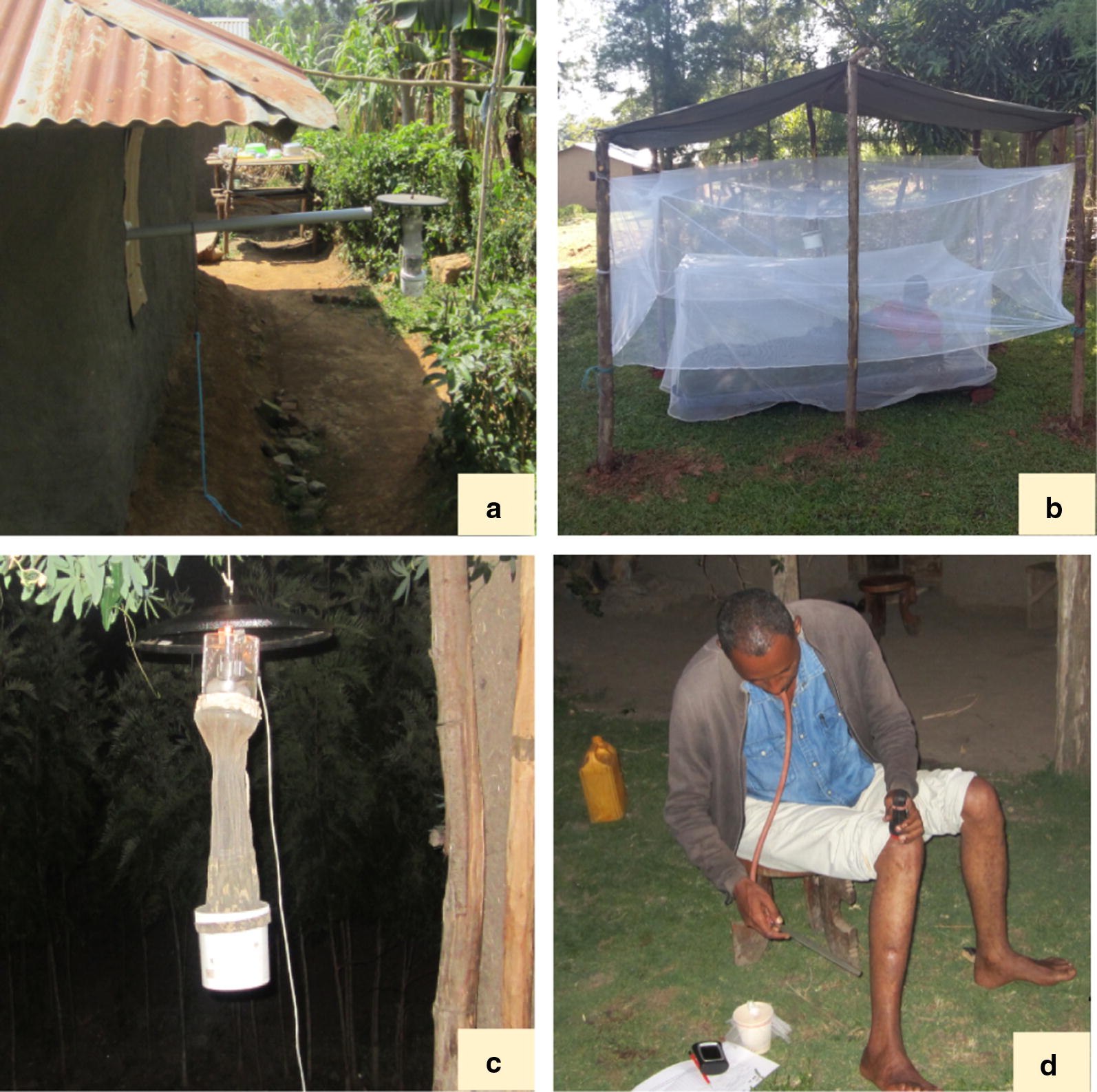
Vector sampling tools [human-odour-baited CDC light trap (**a**), human-baited double net trap (**b**), unbaited CDC light trap (**c**) and human landing catch (**d**)] used for outdoor host-seeking malaria vector surveillance in western Kenya and southwestern Ethiopia

The HBLT comprises a polyvinyl chloride (PVC) pipe that moves human odour from indoor (sleeping room) to outdoor mosquito catching station (Fig. 2a). The inner end of the pipe is wide (4-in. diameter) while its outer segment is narrow (2-in. diameter). A fan was installed into the inner end of the pipe to enhance outflow of the odour. A CDC light trap (John W. Hock Ltd, Gainesville, FL., USA) was set outdoor near the outer end of the pipe to capture mosquitoes attracted to the human odour. The pipe was connected from the sleeping room to the outdoor station through a small hole (2-in. diameter) made on the wall or window of selected houses. The length of the pipe from the wall of the house to its outer end was 2 m. The inner opening of the pipe was covered with untreated net to make sure that the pipe pumps odour only. The inner (wide section) of the pipe was connected with its outer (narrow) section using reducing bush so that the two parts could be easily disconnected when they were not in use. Outdoor host-seeking mosquito collection using the HBLT was done from 18:00 to 6:00 h during each collection night.

#### Human-baited double net trap (HDNT)

The HDNT in this study consisted of two box nets (inner and outer nets) with a roof made of canvas. The inner net (97 cm high × 200 cm long × 100 cm wide) fully protects a human volunteer who rests on a mattress. The outer net (100 cm high × 250 cm long × 150 cm wide) is hung over the inner net and raised 30 cm off the ground. Mosquitoes attracted to the human-bait are collected by setting a CDC light trap between the two nets (Fig. 2b). The HDNT is an exposure-free tool since the lured mosquitoes are captured by the CDC light trap rather than by the person acting as a bait unlike the previously designed bed net traps [29]. Outdoor mosquito sampling using the HDNT was conducted from 18:00 to 6:00 h during each collection night.

#### CDC miniature light traps

Conventional CDC miniature light trap (Fig. 2c) was also set outdoor at about 2 m from each of the selected houses, at a height of 1.5 m from the ground from 18:00 to 06:00 h.

#### Human landing catch (HLC)

The HLC was performed by a male adult volunteer, who acted as both bait and collector (Fig. 2d). The collector seated outdoor on a chair with the legs exposed from foot to knee and captured mosquitoes as soon as they land on the exposed legs before they commence feeding using a flashlight and mouth aspirator [4, 10]. There were two collection shifts: one collector worked from 18:00 to 24:00 h during each collection night, followed by the second collector from 24:00 to 06:00 h. Each hour’s collection was kept separately in labelled paper cups. A supervisor was assigned to coordinate the collection activities and watch volunteers not to fall asleep during the collection nights. All collectors were provided with anti-malaria prophylaxis to avoid a risk of contracting malaria during the collection period. Mosquitoes were identified to species the next morning.

### Experimental design

The study consisted of three consecutive experiments. The first experiment was conducted to compare HBLT with unbaited CDC light trap to test a hypothesis that the use of human-odour in HBLT could significantly improve its trapping efficiency as compared to the unbaited CDC light trap. In the second experiment, HDNT was compared with the HBLT. In the third experiment, the HBLT, HDNT and CDC light trap were compared with HLC, the gold standard method. Details of the experimental designs are presented as follows:

#### Human-odour-baited and unbaited CDC light trap comparison (experiment 1)

This experiment was carried out in Ahero and Iguhu sites, western Kenya. Each study site was classified into three clusters. Two houses with corresponding outdoor mosquito catching stations, about 2 m from each selected house, were selected from each cluster. The HBLT and unbaited CDC light trap were assigned to one of the two outdoor catching stations and swapped between the two houses daily in each cluster in both study sites. The experiment was conducted from November 2015 to February 2016. A total of 60 trapping nights were done for each trap in each study site.

#### Human-odour-baited CDC light trap and human-baited double net trap comparison (experiment 2)

Experiment 2 was conducted from June to July 2017 in the same study sites as experiment 1, using the same two houses in each cluster. The HBLT and HDNT were assigned to one of the two outdoor catching stations and swapped between the two houses daily in each cluster in both study sites. A total of 42 trapping nights were done for each trapping method in each study site.

#### Comparison of alternative outdoor traps with human landing catch (experiment 3)

The third experiment was conducted in Bulbul, southwestern Ethiopia. Four representative houses of similar size and design with corresponding outdoor catching stations were randomly selected. The HBLT, HDNT, CDC light trap and HLC were assigned to one of the four outdoor catching stations. The traps were rotated among the selected houses once monthly using 4 × 4 Latin Square Design. All traps were set simultaneously from 18:00 to 6:00 h. A total of 48 trapping nights were conducted for each trapping method. The experiment was conducted from January to December 2018.

### Sample processing

All collected mosquito samples were identified morphologically to species or species complexes using morphological identification keys [46]. Adult female *Anopheles* mosquitoes were kept individually in labelled 1.5 ml Eppendorf tubes containing silica gel desiccant. Samples were stored at − 20 °C freezer at Climate and Human Health Research Laboratory of Kenya Medical Research Institute (KEMRI) or Jimma University Tropical and Infectious Diseases Research Center (TIDRC) Laboratory until used for further processing.

### Identification of vector species complexes

All *An. gambiae* sensu lato (s.l.) samples collected from Iguhu, and sub-samples of *An. gambiae* s.l. randomly selected from each trap for Ahero and Bulbul sites were analysed by polymerase chain reaction (PCR) for identification of their sibling species, following the protocol developed by Scott et al. [47]. Moreover, sub-samples of *An. funestus* s.l. collected from Ahero and Iguhu sites were identified to sibling species by PCR following the protocol developed by Koekemoer et al. [48].

### Detection of sporozoite infections

Dried head and thorax of the preserved *Anopheles* mosquito specimens were carefully separated from the abdomen and tested for *P. falciparum* and *P. vivax* circum-sporozoite protein (CSP) using sand-witch ELISA method [49, 50].

### Data analysis

The difference in *Anopheles* mosquito density among different trapping methods was compared using generalized linear model based on a negative binomial distribution. Trap type was fitted as the main factor in the model. Experimental night was treated as a covariate for the first and second experiments, whereas sampling month was also considered as a covariate for the third experiment. The estimated marginal mean (EMM) density of *Anopheles* mosquitoes was determined for each trap using the negative binomial regression by adjusting for experimental night and month. Gini-Simpson’s diversity index (1-D) [51–53] was applied to evaluate mosquito species diversity for each trap. To determine the statistical significance of difference in species diversity among the traps, 95% confidence intervals (CI) were calculated [54]. The Simpson’s index of evenness (E) was computed to obtain a measure of the relative abundance of different mosquito species in each trapping method [51, 55].

Further analysis was conducted for the third experiment to determine whether each of the alternative outdoor trapping methods was correlated with the reference method i.e. HLC. Pearson correlation coefficient for the relationship among log-transformed catches for each *Anopheles* species was determined. To test if the sampling efficiency of each alternative trap (HBLT, HDNT or CDC light trap) relative to the HLC was affected by mosquito density, the ratios of the number of mosquitoes in each alternative trap to the number of mosquitoes in HLC [log(HLC + 1) − log(Alternative trap + 1)] were plotted against the average mosquito abundance, calculated as [log(HLC + 1) + log(Alternative trap + 1)]/2 [56]. Simple linear regression analysis was done for the relationship between the ratios and their average mosquito abundance [56]. The value of R-square (*R*^2^) derived from the analysis was then interpreted as an estimate of the proportion of deviation from perfect linear correlation due to density-dependence rather than random error, with a high and significant value indicating density-dependence.

The sporozoite rate was estimated as the proportion of mosquitoes positive for *Plasmodium* CSP over the total number tested. Data were analysed using SPSS version 20.0 (SPSS, Chicago, IL, USA) software package. p < 0.05 was considered statistically significant during the analysis.

## Results

### Mosquito species composition and abundance

Overall, 30,278 female mosquitoes (25,135 from Ahero, 1407 from Iguhu and 3736 from Bulbul) were collected outdoors over the course of 600 trapping nights. Of these, 16,963 (56.0%) were anophelines, with the remaining 13,315 (44.0%) being *Culex* species. 15,201 of the anophelines were collected from Ahero and Iguhu sites (5042 by HBLT, 1128 by CDC light trap and 9031 by HDNT). *Anopheles gambiae* s.l. was the predominant species, accounting for 57.3% of the anophelines collected from Ahero and Iguhu, followed by *An. pharoensis* (22.3%), *An. coustani* (15.5%) and *An. funestus* s.l. (4.9%). In Bulbul site, *An. pharoensis* was the most abundant species, accounting for 41.0% of the collected anophelines, followed by *An. coustani* (30.7%), *An. gambiae* s.l. (27.7%), *An. squamosus* (0.4%) and *An. funestus* s.l. (0.2%).

### Composition of vector species complexes

A total of 602 *An. gambiae* s.l. specimens [258 from Ahero, 184 from Iguhu and 160 from Bulbul] and 90 *An. funestus* s.l. (from Ahero and Iguhu) were analysed for identification of sibling species. Of these, 552 *An. gambiae* s.l. and 84 *An. funestus* s.l. specimens were successfully amplified and identified to species by PCR. In Ahero, all of the amplified *An. gambiae* s.l. specimens were confirmed to be *An. arabiensis*. In Iguhu, *An. arabiensis* and *An. gambiae* s.s. accounted for 15.7 and 84.3% of the *An. gambiae* s.l., respectively. The sibling species composition of *An. gambiae* s.l. did not vary among the different trapping methods (*χ*^2^ = 0.086, *df* = 2, p = 0.958). Of the amplified *An. funestus* s.l. specimens, *An. funestus* s.s. and *Anopheles leesoni* accounted for 90.5 and 9.5%, respectively. Similar to Ahero, *An. arabiensis* was the only identified member species of the *An. gambiae* s.l. in Bulbul site.

### Mosquito density and species diversity

#### Human-odour-baited and unbaited CDC light trap comparison (experiment 1)

Between November 2015 to February 2016, a total of 2783 female *Anopheles* mosquitoes were collected by HBLT and CDC light trap in Ahero and Iguhu sites. Overall, HBLT yielded 1.43 (95% CI 1.09–1.86, p = 0.009) times higher density of anophelines than CDC light trap (Table 1). In Ahero, HBLT caught 2.23 (95% CI 1.49–3.36, p < 0.001) times as many *An. arabiensis* per night as CDC light trap. Similarly, HBLT captured 2.11 (95% CI 1.28–3.47, p = 0.003) times higher number of *An. funestus* s.l. per night compared to CDC light trap. There was no significant difference between HBLT and CDC light trap in terms of the mean density of *An. pharoensis* and *An. coustani* (p > 0.05). In Iguhu site, the density of anophelines was low from both HBLT and CDC light trap (Table 1).

**Table 1:** Estimates of a negative binomial regression for the comparison of outdoor host-seeking anopheline density between HBLT and CDC light trap in Ahero and Iguhu, western Kenya

Site and species	Traps	Number collected	EMM (95% CI)	OR (95% CI)	p value
Ahero					
*An. arabiensis*	HBLT	332	5.52 (4.19–7.26)	2.23 (1.49–3.36)	< 0.001*
	Light trap	149	2.47 (1.83–3.33)	1.0^a^	
*An. funestus* s.l.	HBLT	99	1.65 (1.20–2.27)	2.11 (1.28–3.47)	0.003*
	Light trap	47	0.78 (0.53–1.15)	1.0^a^	
*An. pharoensis*	HBLT	554	8.21 (6.27–10.75)	1.28 (0.87–1.87)	0.213
	Light trap	421	6.43 (4.89–8.46)	1.0^a^	
*An. coustani*	HBLT	641	9.06 (6.93–11.86)	1.16 (0.79–1.71)	0.442
	Light trap	497	7.80 (5.95–10.23)	1.0^a^	
Iguhu					
*An. gambiae* s.l.	HBLT	15	0.22 (0.12–0.41)	2.10 (0.79–5.57)	0.137
	Light trap	7	0.11 (0.05–0.24)	1.0a	
*An. funestus* group	HBLT	10	0.16 (0.08–0.31)	1.65 (0.56–4.87)	0.360
	Light trap	6	0.10 (0.04–0.22)	1.0^a^	
*An. coustani*	HBLT	4	0.07 (0.02–0.18)	4.0 (0.43–36.94)	0.221
	Light trap	1	0.02 (0.002–0.12)	1.0^a^	
Total anophelines	HBLT	1655	12.74 (10.58–15.35)	1.43 (1.09–1.86)	0.009*
	Light trap	1128	8.92 (7.38–10.78)	1.0^a^	

A total of 60 trap-nights were conducted for each trap in each study site

*HBLT* human odour-baited CDC light trap, *EMM* estimated marginal mean density, *OR* odds ratio, *CI* confidence interval

* Statistically significant

^a^Reference value

The diversity of mosquito species captured was significantly higher for HBLT (Simpson diversity index ± 2SD = 0.63 ± 0.01) than for CDC light trap (0.59 ± 0.02). Moreover, the HBLT collected mosquitoes of different species more homogenously (Simpson evenness, E = 0.79 ± 0.02) than CDC light trap (0.71 ± 0.02).

#### Human-odour-baited CDC light trap and human-baited double net trap comparison (experiment 2)

A total of 12,418 *Anopheles* mosquitoes were collected by HBLT and HDNT in Ahero and Iguhu sites during the second experiment. Overall, HDNT yielded 2.75 (95% CI 2.01–3.74, p < 0.001) times higher density of anophelines compared to HBLT (Table 2). In Ahero, HDNT caught 3.43 (95% CI 2.22–5.30, p < 0.001) times as many *An. arabiensis* per night as HBLT. Likewise, HDNT captured 3.24 (95% CI 1.99–5.25, p < 0.001) times as many *An. funestus* s.l. and 3.55 (95% CI 2.25–5.61, p < 0.001) times as many *An. coustani* per night as HBLT. No significant difference was found in the mean density of *An. pharoensis* between the two traps (p = 0.183). In Iguhu site, the mean density of *An. gambiae* s.l. and *An. funestus* s.l. did not vary significantly between HDNT and HBLT (p > 0.05) (Table 2).

**Table 2:** Estimates of a negative binomial regression for the comparison of outdoor host-seeking anopheline density between HDNT and HBLT in Ahero and Iguhu, western Kenya

Site and species	Traps	Number collected	EMM (95% CI)	OR (95% CI)	p value
Ahero					
*An. arabiensis*	HDNT	6188	148.83 (109.67–201.97)	3.43 (2.22–5.30)	< 0.001*
	HBLT	1862	43.40 (31.90–59.04)	1.0^a^	
*An. funestus* s.l.	HDNT	392	9.21 (6.67–12.71)	3.24 (1.99–5.25)	< 0.001*
	HBLT	137	2.84 (1.99–4.06)	1.0^a^	
*An. pharoensis*	HDNT	1386	32.91 (24.09–44.96)	1.36 (0.87–2.13)	0.183
	HBLT	1016	24.25 (17.72–33.19)	1.0^a^	
*An. coustani*	HDNT	895	21.30 (15.59–29.11)	3.55 (2.25–5.61)	< 0.001*
	HBLT	252	6.00 (4.32–8.34)	1.0^a^	
Iguhu					
*An. gambiae* s.l.	HDNT	92	2.17 (1.50–3.13)	1.29 (0.75–2.20)	0.353
	HBLT	70	1.68 (1.14–2.47)	1.0^a^	
*An. funestus* s.l.	HDNT	34	0.81 (0.52–1.27)	1.42 (0.72–2.79)	0.308
	HBLT	24	0.57 (0.35–0.94)	1.0^a^	
*An. pharoensis*	HDNT	6	0.13 (0.05–0.32)	1.45 (0.38–5.58)	0.587
	HBLT	4	0.09 (0.03–0.26)	1.0^a^	
*An. coustani*	HDNT	38	0.86 (0.55–1.34)	1.65 (0.83–3.27)	0.151
	HBLT	22	0.52 (0.31–0.87)	1.0^a^	
Total anophelines	HDNT	9031	108.69 (87.54–134.96)	2.75 (2.01–3.74)	< 0.001*
	HBLT	3387	39.60 (31.84–49.25)	1.0^a^	

A total of 42 trap-nights were conducted for each trap in each study site

*HBLT* human-odour-baited CDC light trap, *HDNT* human-baited double net trap, *EMM* estimated marginal mean density, *OR* odds ratio, *CI* confidence interval

* Statistically significant

^a^Reference value

The diversity of mosquito species collected did not vary significantly between HDNT (Simpson diversity index = 0.66 ± 0.01) and HBLT (0.64 ± 0.01). Similarly, the species evenness did not vary significantly between the HDNT (E = 0.82 ± 0.01) and HBLT (0.81 ± 0.01).

#### Comparison of alternative outdoor traps with human landing catch (experiment 3)

A total of 1762 *Anopheles* mosquitoes were caught outdoors by HBLT, HDNT, CDC light trap and HLC in Bulbul site from January to December 2018. The EMM density of each anopheline species per trap is shown in Table 3. On average, HBLT caught 2.19 (95% CI 1.18–4.10, p 0.014) times as many *An. arabiensis* per night as CDC light trap, while HDNT caught 6.53 (95% CI 3.64–11.72, p < 0.001) times as many *An. arabiensis* per night as CDC light trap. The mean density of *An. arabiensis* did not vary between HDNT and HLC (p = 0.098), whereas the HLC caught 4.35 (95% CI 2.64–7.17, p < 0.001) times as many *An. arabiensis* as HBLT and 9.54 (95% CI 5.35–17.02, p < 0.001) times as many as CDC light trap.

**Table 3:** Estimates of a negative binomial regression for comparison of outdoor host-seeking anopheline density between different traps in Bulbul, southwestern Ethiopia

Site and species	Traps	Number collected	EMM (95% CI)	OR (95% CI)	p value
*An. arabiensis*	HBLT	55	1.12 (0.76–1.65)	0.23 (0.14–0.38)	< 0.001*
	HDNT	168	3.32 (2.40–4.59)	0.69 (0.44–1.07)	0.098
	Light trap	25	0.51 (0.31–0.83)	0.11 (0.06–0.19)	< 0.001*
	HLC	240	4.85 (3.56–6.63)	1.0^a^	
*An. pharoensis*	HBLT	78	1.47 (1.02–2.12)	0.20 (0.13–0.33)	< 0.001*
	HDNT	243	4.79 (3.51–6.55)	0.66 (0.43–1.02)	0.062
	Light trap	35	0.72 (0.46–1.12)	0.10 (0.06–0.17)	< 0.001*
	HLC	366	7.25 (5.35–9.81)	1.0^a^	
*An. coustani*	HBLT	52	1.01 (0.67–1.51)	0.15 (0.09–0.25)	< 0.001*
	HDNT	101	1.83 (1.29–2.61)	0.28 (0.17–0.44)	< 0.001*
	Light trap	26	0.48 (0.29–0.78)	0.07 (0.04–0.13)	< 0.001*
	HLC	362	6.62 (4.88–18.99)	1.0^a^	
Other anophelines^b^	HBLT	2	0.04 (0.01–0.16)	0.35 (0.07–1.83)	0.213
	HDNT	3	0.06 (0.02–0.19)	0.52 (0.12–2.21)	0.372
	Light trap	0	0	NA	NA
	HLC	6	0.12 (0.04–0.27)	1.0^a^	
Total anophelines	HBLT	187	3.63 (2.63–5.00)	0.19 (0.12–0.29)	< 0.001*
	HDNT	515	10.02 (7.45–13.49)	0.53 (0.35–0.80)	0.003*
	Light trap	86	1.74 (1.21–2.48)	0.09 (0.06–0.15)	< 0.001*
	HLC	974	18.99 (14.20–25.40)	1.0^a^	

A total of 48 trap-nights were conducted for each trap in each study site

*HBLT* human-odour-baited CDC light trap, *HDNT* human-baited double net trap, *HLC* human landing catch, *EMM* estimated marginal mean density, *OR* odds ratio, *CI* confidence interval

* Statistically significant

^a^Reference value

^b^Other anophelines include *An. squamosus* and *An. funestus* s.l.

The mean density of *An. pharoensis* captured by HBLT was 2.04 (95% CI 1.15–3.61, p = 0.015) times higher compared to CDC light trap, whereas the mean density of the same species collected by HDNT was 6.65 (95% CI 3.87–11.42, p < 0.001) times higher compared to the CDC light trap. No significant difference was found in the mean density of *An. pharoensis* between HDNT and HLC (p = 0.062), while the HLC collected 4.94 (95% CI 3.07–7.95, p < 0.001) times as many *An. pharoensis* per night as HBLT and 10.06 (95% CI 5.89–17.18, p < 0.001) times as many as CDC light trap (Table 3).

The mean density of *An. coustani* caught by HBLT was 2.11 (95% CI 1.12–3.99, p = 0.021) times higher compared to CDC light trap, while the mean density of *An. coustani* caught by HDNT was 3.84 (95% CI 2.10–7.02, p < 0.001) times higher compared to CDC light trap. The HLC captured 3.61 (95% CI 2.26–5.76, p < 0.001) times as many *An. coustani* per night as HDNT, 6.57 (95% CI 3.95–10.90) times as many as HBLT and 13.88 (95% CI 7.79–24.72, p < 0.001) times as many as CDC light trap. Very few *An. squamosus* and *An. funestus* s.l. were collected by HLC, HDNT and HBLT, whereas none of this species were collected by CDC light trap (Table 3).

The diversity of mosquito species collected in Bulbul was significantly higher for HDNT (Simpson diversity index = 0.70 ± 0.01) than for HBLT (0.63 ± 0.04), CDC light trap (0.50 ± 0.07) and HLC (0.63 ± 0.02). The diversity of mosquito species collected by HBLT was significantly higher than that of CDC light trap, whereas the HBLT and HLC collected mosquito of similar species diversity. The HDNT collected mosquitoes of different species more homogeneously (E = 0.85 ± 0.02) than HBLT (E = 0.76 ± 0.05), CDC light trap (E = 0.67 ± 0.09) and HLC (E = 0.75 ± 0.02).

### Correlation of the alternative traps with human landing catch

**Fig. 3:**
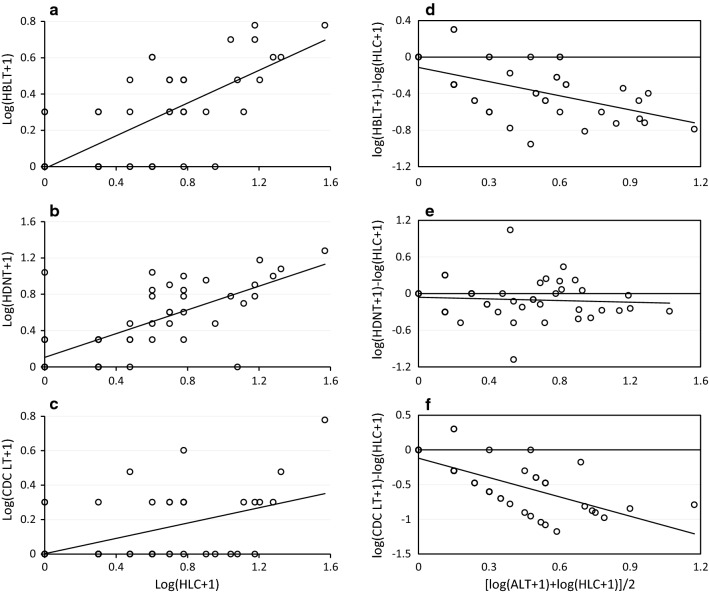
Correlation and density-dependence of the alternative outdoor trapping methods relative to human landing catch for catching *Anopheles* mosquitoes in Bulbul, southwestern Ethiopia [Correlation of human-odour-baited CDC light trap (**a**), human-baited double net trap (**b**) and unbaited CDC light trap (**c**) with human landing catch. The sampling efficiency (RSE) of human-odour-baited CDC light trap (**d**), human-baited double net trap (**e**) and unbaited CDC light trap (**f**) relative to human landing catch]. ALT represents alternative traps

The correlation coefficients of alternative traps with HLC are shown in Table 4. There were significant positive correlations between HDNT and HLC in terms of the number of *An. arabiensis* (r = 0.691, p = < 0.001) and *An. pharoensis* (0.739, p < 0.001) (r = 0.691, p = < 0.001) captured, and *R*^2^ values did not deviate significantly from zero (Fig. 3; Table 4), which means that the relative sampling efficiency (RSE) of the HDNT was not dependent on mosquito density for these species. For *An. coustani*, a significant positive correlation was found between the HDNT and HLC (r = 0.655, p < 0.001), but the RSE was density-dependent. Significant positive correlations were also found between HBLT and HLC for *An. arabiensis* (r = 0.708, p < 0.001), *An. pharoensis* (r = 0.454, p = 0.001) and *An. coustani* (r = 0.664, p = 0.001), but the RSEs were dependent on mosquito density (Fig. 3; Table 4).

**Table 4:** Correlation and density-dependence of the sampling efficiency of alternative outdoor trapping methods relative to human landing catch in Bulbul, southwestern Ethiopia

Species	Alternative vs. HLC	Correlation coefficient		Density-dependence		
		R	p*-*value	R-square	T	p-value
*An. arabiensis*	HBLT	0.708	< 0.001	0.304	20.135	< 0.001
	HDNT	0.691	< 0.001	0.006	0.284	0.597
	Light trap	0.469	0.001	0.461	39.408	< 0.00
*An. pharoensis*	HBLT	0.454	0.001	0.140	7.505	0.009
	HDNT	0.739	< 0.001	0.066	3.244	0.078
	Light trap	0.199	0.176	0.411	32.042	< 0.001
*An. coustani*	HBLT	0.664	< 0.001	0.521	50.020	< 0.001
	HDNT	0.655	< 0.001	0.233	13.973	0.001
	Light trap	0.569	< 0.001	0.657	88.070	< 0.001

*HBLT* human-odour-baited CDC light trap, *HDNT* human-baited double net trap, *HLC* human landing catch

### Sporozoite rate

Overall, 7344 (43.3% of the total) *Anopheles* mosquitoes (5273 from Ahero, 309 from Iguhu and 1762 from Bulbul) were tested for *P. falciparum* and *P. vivax* CSP. Of these, 27 specimens (17 from Ahero, 4 from Iguhu and 6 from Bulbul) were positive for *Plasmodium* CSP.

Table 5 shows the sporozoite rates of anophelines collected from Ahero and Iguhu sites. In Ahero, the sporozoite rate of *An. arabiensis* was 0.12% from HBLT and 0.16% from HDNT. None of the tested *An. arabiensis* from CDC light trap were positive. In the same study site, the sporozoite rate of *An. funestus* s.l. was 2.1% from HBLT, 2.4% from HDNT and 2.1% from CDC light trap. In Iguhu, the sporozoite rate of *An. gambiae* s.s. was 1.5% from HBLT and 2.9% from HDNT, while the sporozoite rate of *An. funestus* s.l. from HDNT was 3.0%. No CSP was detected in *An. funestus* s.l. collected by HBLT and CDC light trap. Thus, the overall sporozoite rate of *An. arabiensis, An. gambiae* s.s. and *An. funestus* s.l. was 0.14, 2.1 and 2.2%, respectively. None of the tested *An. pharoensis* and *An. coustani* specimens were positive.

**Table 5:** *Plasmodium falciparum* sporozoite rates of outdoor host-seeking *Anopheles* mosquitoes collected by different trapping methods in Ahero and Iguhu, western Kenya

Study site and species	Parameters	Experiment 1		Experiment 2		Total
		HBLT	Light trap	HBLT	HDNT	
Ahero						
*An. arabiensis*	No tested	201	149	651	1929	2930
	Pf +ve (%)	0	0	1 (0.15)	3 (0.16)	4 (0.14)
*An. funestus* s.l.	No tested	99	47	136	287	570
	Pf +ve (%)	2 (2.0)	1 (2.1)	3 (2.2)	7 (2.4)	13 (2.3)
*An. pharoensis*	No tested	168	146	305	416	1035
	Pf +ve (%)	0	0	0	0	0
*An. coustani*	No tested	193	150	125	270	738
	Pf +ve (%)	0	0	0	0	0
Iguhu						
*An. gambiae* s.s.	No tested	12	6	53	69	140
	Pf +ve (%)	0	0	1 (1.9)	2 (2.9)	3 (2.1)
*An. funestus* s.l.	No tested	9	6	20	33	68
	Pf +ve (%)	0	0	0	1 (3.0)	1 (1.5)
*An. arabiensis*	No tested	2	1	11	12	26
	Pf +ve (%)	0	0	0	0	0
*An. pharoensis*	No tested	0	0	4	6	10
	Pf +ve (%)	0	0	0	0	0
*An. coustani*	No tested	4	1	22	38	65
	Pf +ve (%)	0	0	0	0	0

HBLT: human-odour-baited CDC light trap; HDNT: human-baited double net trap; *Pf* +ve: number of *P. falciparum* positive *Anopheles* mosquitoes (rate in percent)

In Bulbul site, of the assayed anopheline specimens, 6 (2 *An. arabiensis*, 3 *An. pharoensis* and 1 *An. coustani*) were positive for *Plasmodium* CSP (four specimens for *P. vivax* and two for *P. falciparum*) (Table 6). The sporozoite rate of *An. arabiensis* was 0.6% from HDNT and 0.4% from HLC. No CSP was detected in *An. arabiensis* collected by HBLT and CDC light trap. The sporozoite rate of *An. pharoensis* was 1.3% from HBLT, 0.4% from HDNT and 0.3% from HLC. The sporozoite rate of *An. coustani* from HLC was 0.3%, whereas no CSP was detected in *An. coustani* collected by the other trapping methods. Hence, the overall sporozoite rate of *An. arabiensis*, *An. pharoensis* and *An. coustani* was 0.4, 0.3 and 0.2%, respectively.

**Table 6:** Sporozoite rates of outdoor host-seeking *Anopheles* mosquitoes collected by different methods in Bulbul, southwestern Ethiopia

Method	Species	No tested	Pf n (%)	Pv210 n (%)	Pv247 n (%)	Total n (%)
HBLT	*An. arabiensis*	55	0	0	0	0
	*An. pharoensis*	78	0	1 (1.3)	0	1 (1.3)
	*An. coustani*	52	0	0	0	0
	*An. squamosus*	1	0	0	0	0
	*An. funestus* s.l.	1	0	0	0	0
HDNT	*An. arabiensis*	168	1 (0.6)	0	0	1 (0.6)
	*An. pharoensis*	243	0	0	1 (0.4)	1 (0.4)
	*An. coustani*	101	0	0	0	0
	*An. squamosus*	2	0	0	0	0
	*An. funestus* s.l.	1	0	0	0	0
Light trap	*An. arabiensis*	25	0	0	0	0
	*An. pharoensis*	35	0	0	0	0
	*An. coustani*	26	0	0	0	0
HLC	*An. arabiensis*	240	1 (0.4)	0	0	0 (0.4)
	*An. pharoensis*	366	0	1 (0.3)	0	1 (0.3)
	*An. coustani*	362	0	1 (0.3)	0	1 (0.3)
	*An. squamosus*	4	0	0	0	0
	*An. funestus* s.l.	2	0	0	0	0
Overall	*An. arabiensis*	488	2 (0.4)	0	0	2 (0.4)
	*An. pharoensis*	722	0	2 (0.3)	1 (0.1)	3 (0.4)
	*An. coustani*	541	0	1 (0.2)	0	1 (0.2)
	*An. squamosus*	7	0	0	0	0
	*An. funestus* s.l.	4	0	0	0	0

HBLT: human-odour-baited CDC light trap; HDNT: human-baited double net trap; HLC: human landing catch; *Pf: P. falciparum*; *Pv: P. vivax*; n: number positive (rate in percent)

## Discussion

In this study, the potential of two human-odour baited traps, the HBLT and HDNT, to provide exposure-free alternatives to the HLC and CDC light trap for surveillance of outdoor host-seeking African malaria vectors was evaluated. The results showed that both HBLT and HDNT yielded significantly higher anopheline mosquito density compared to conventional CDC light trap. This suggests that the use of human-bait in HBLT and HDNT significantly enhanced the trapping efficiency of both traps. Moreover, the HDNT yielded a similar vector density as HLC. This indicates the usefulness of these tools for outdoor host-seeking vector surveillance.

The HBLT collected about twice as many *An. arabiensis* and *An. funestus* s.l. as unbaited CDC light trap. This indicates that the HBLT may also surpass the trapping efficiency of CO_2_-baited CDC light traps that have been compared with unbaited CDC light traps previously [57–60]. For instance, CO_2_-baited CDC light trap captured 1.39 times as many anophelines as unbaited CDC light trap in Thailand [57], whereas in other studies conducted in south-central Ethiopia and Suriname, synthetic CO_2_ did not improve the trapping efficiency of CDC light traps [28, 58]. The lower sampling efficiency of the CO_2_-baited CDC light traps in the previous studies might be due to a lower attraction of synthetic CO_2_ as compared to natural human odour. It was hypothesized that when synthetic CO_2_ is used in traps in isolation from other attractant stimuli produced by hosts, it could be considered as an artificial arrangement, and mosquitoes might not fly directly towards it but rather show an erratic behaviour [4]. Thus, the HBLT could represent a better outdoor vector surveillance tool than both unbaited and CO_2_-baited CDC light traps.

However, the HBLT yielded 4.35 times lower number of *An. arabiensis* compared to HLC, and 4.94 and 6.57 times lower for *An. pharoensis* and *An. coustani*, respectively. Similarly, the HBLT yielded significantly lower density of anophelines than HDNT. These variations are probably due to the difference in the location of persons used as bait. Although all traps were set outdoors in this study, a bait for HBLT was located indoor and odour was pumped-out through a pipe, while in the case of HLC and HDNT, human-baits were positioned outdoors on the actual mosquito catching stations. This means that the HBLT lacks thermal cues that may serve as supplementary short-range mosquito attractant [60], unlike the HLC and HDNT. On the other hand, HLC may also overestimate human-biting rates to some extent since the human-baits are relatively more available to host-seeking mosquitoes than under normal circumstance. Although it is habitual practice in Africa to spend evening and early-morning hours outdoors [61–63], people may not stay undisturbed in one place with legs exposed throughout the night unlike that of HLC.

The HDNT caught 6.53 times as many *An. arabiensis* and 6.65 times as many *An. pharoensis* as CDC light trap in Bulbul while the mean density of both *An. arabiensis* and *An. pharoensis* did not vary significantly between the HDNT and HLC, indicating the potential of the HDNT to substitute HLC. In previous studies in Africa, in which human served as both bait and collector in double net traps, the double net traps yielded significantly lower number of anophelines than HLC [31, 64]. The double net trap collected 7.5 times lower number of anophelines compared to HLC in Cameroon [31] and about four times lower number of anophelines in Nigeria [64]. The double net traps might have underestimated the density of anophelines in the previous studies since mosquitoes could escape the double net traps when they were unable to reach the bait [4]. While the probability of mosquitoes escaping the double net traps could be minimized by conducting hourly collections as described by Tangena et al. [29], such approach may also expose humans to infective mosquito bites when they get out of the inner net to perform mosquito collection. In the present study, the trapping efficiency of HDNT was enhanced by setting a CDC light trap between the double nets so that mosquitoes could be trapped as soon as they enter the HDNT. The HDNT could also provide a full protection since a person serving as bait in the HDNT is not involved in mosquito collection.

Moreover, HDNT showed significant positive correlation with HLC for sampling *An. arabiensis* and other secondary vectors, and its sampling efficiency did not depend on mosquito density. This suggests that the HDNT could represent an efficient alternative tool to HLC for surveillance of outdoor host-seeking malaria vectors. Furthermore, the HDNT collected higher mosquito species diversity compared to both CDC light trap and HLC. This makes the HDNT more useful for exploring outdoor mosquito species diversity.

The advantage of HDNT and HBLT is that they are not as labour intensive as HLC. In HDNT, a person acting as bait can rest throughout the night. Similarly, HBLT uses odours from human resting in ordinary sleeping room. In the case of HLC [65] and the previous design of double bed net traps [4, 29, 30, 66], the collectors have to remain active, and collect mosquitoes throughout the night. In addition, mosquito collections using HDNT and HBLT do not rely on the skill of collectors unlike that of HLC which is prone to bias due to interpersonal variation in the skill of the collectors.

Both HBLT and HDNT have limitations. The HBLT uses two batteries, one for a CDC light trap and the other for a pipe, hence may not be feasible in settings where there is no easy access to electricity. Using human odour in HBLT requires connecting a pipe from a sleeping room to outdoor mosquito catching station through a hole made on window or mud-wall of the room. Rooms with cement-plastered wall and without window are not appropriate to set the HBLT. Hence, further modification is needed to easily dispense human odour. Both HBLT and HDNT were set in the evening and trapped mosquitoes were collected from the traps once in the morning instead of hourly collection, hence hourly anopheline mosquito densities were not compared between these traps and HLC. Further modification using collection bottle rotator that allows automatic hourly collections may be needed to use them for monitoring vector biting times.

## Conclusion

This study revealed that both HBLT and HDNT performed better than conventional CDC light traps to sample outdoor host-seeking malaria vectors. Moreover, the HDNT yielded a similar vector density as outdoor HLC, suggesting that it could represent an alternative tool to HLC for outdoor biting malaria vector surveillance. The HBLT could be used as an alternative when the HDNT cannot be used especially when there is flood that may affect a person resting under the net.

## Data Availability

Data supporting the conclusions of this article are included within the article. Raw data are available from the corresponding author upon reasonable request.

## Abbreviations

CDC: Centers for Disease Control and Prevention
CI: Confidence interval
CSP: Circumsporozoite protein
EIR: Entomological inoculation rate
ELISA: Enzyme-linked immunosorbent assay
EMM: Estimated marginal mean density
HBLT: Human-odour-baited CDC light trap
HBR: Human-biting rate
HDNT: Human-baited double net trap
HLC: Human landing catch
OR: Odds ratio
PCR: Polymerase chain reaction
RSE: Relative sampling efficiency
